# Cerebral autoregulation in paediatric and neonatal intensive care: A scoping review

**DOI:** 10.1177/0271678X241261944

**Published:** 2024-06-13

**Authors:** Marta Fedriga, Silvia Martini, Francesca G Iodice, Cristine Sortica da Costa, Stefano Pezzato, Andrea Moscatelli, Erta Beqiri, Marek Czosnyka, Peter Smielewski, Shruti Agrawal

**Affiliations:** 1 Neonatal and Paediatric Intensive Care Unit, 18572 IRCCS Giannina Gaslini Institute, Genoa, Italy; 2 Neonatal Intensive Care Unit, IRCCS AOUBO, Department of Medical and Surgical Sciences, University of Bologna, Bologna, Italy; 3 Paediatric Cardiac Anaesthesia and Intensive Care Unit, IRCCS, Bambino Gesu’ Hospital, Rome, Italy; 4 Neonatal Intensive Care Unit, Great Ormond Street Hospital, London, UK; 5 Brain Physics Laboratory, Division of Neurosurgery, Department of Clinical Neurosciences, 2152 University of Cambridge, UK; 6 Department of Paediatric Intensive Care, 2152 Addenbrooke’s Hospital, University of Cambridge, Cambridge, UK

**Keywords:** Cerebral autoregulation, cerebrovascular reactivity, paediatric intensive care, neonatal intensive care, pressure reactivity

## Abstract

Deranged cerebral autoregulation (CA) is associated with worse outcome in adult brain injury. Strategies for monitoring CA and maintaining the brain at its ‘best CA status’ have been implemented, however, this approach has not yet developed for the paediatric population. This scoping review aims to find up-to-date evidence on CA assessment in children and neonates with a view to identify patient categories in which CA has been measured so far, CA monitoring methods and its relationship with clinical outcome if any. A literature search was conducted for studies published within 31st December 2022 in 3 bibliographic databases. Out of 494 papers screened, this review includes 135 studies. Our literature search reveals evidence for CA measurement in the paediatric population across different diagnostic categories and age groups. The techniques adopted, indices and thresholds used to assess and define CA are heterogeneous. We discuss the relevance of available evidence for CA assessment in the paediatric population. However, due to small number of studies and heterogeneity of methods used, there is no conclusive evidence to support universal adoption of CA monitoring, technique, and methodology. This calls for further work to understand the clinical impact of CA monitoring in paediatric and neonatal intensive care.

## Introduction

**Figure 1.**
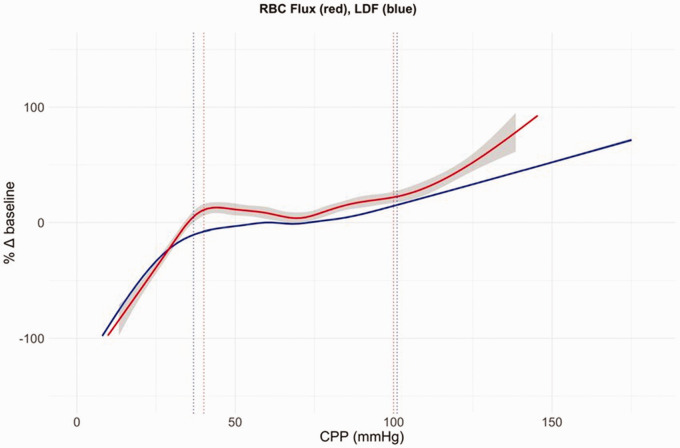
Autoregulatory curve. Autoregulation curve combined changes in LDF (laser doppler flow) (blue) and RBC (red blood cells) flux (red) for 10 hypotensive and 10 hypertensive piglet experiments plotted against CPP. Grey shading represents the standard error (for the LDF data curve there was no visible standard error). Dotted lines show the mean lower and upper limits of autoregulation.^27^

Adaptive changes in the tone of cerebral vessels in response to a pressure stimulus define cerebral autoregulation (CA).^1,2^ This complex phenomenon reflects brain’s ability to maintain an adequate cerebral blood flow (CBF) irrespective of changes in systemic blood pressure or cerebral perfusion pressure (CPP).^3^ In 1938, Mogens Fog first described the relationship between arterial blood pressure (ABP) and the diameter of pial arterioles, suggesting a decrease and widening in the arteriolar diameter with ABP increase and decrease respectively.^4^ In 1959, Niel Lassen postulated the existence of an autoregulatory plateau where CBF remained constant despite changing ABP, and plotted the so-called “Lassen-curve”.^5^ This paved the path to the concepts of lower (LLA) and upper limits of autoregulation (ULA) beyond which CA is no longer effective.^6^ Although the Lassen-curve has since been challenged, overall concept of the autoregulatory plateau has stood the test of time with improved understanding of individual differences^7^ (Figure 1).

CA can be damaged in various neurological conditions like traumatic brain injury (TBI), hypoxic ischaemic neurological insult, intracranial bleed, stroke and so on, and this derangement can alter clinical outcomes.^8–10^ If CA is impaired, CBF becomes pressure passive exposing the brain to ischaemic or hyperaemic insults depending on the ABP changes. Accumulating evidence supports the significant relationship between time spent with impaired CA and neurodevelopmental outcomes.^11,12^ In the paediatric population, developmental trajectory of the CA mechanisms across paediatric ages pose further challenges.^13^ Though age related differences have not been fully characterized, it would be interesting to investigate this field to find out variations in cerebrovascular physiology or physiopathology as previously shown in animal studies.^14^ Therefore, a better understanding of CA can give useful information to titrate treatments and improve outcomes in infants and children at risk of or with brain injury.^15^

Various methods are used to assess CA based on the fundamental principle that if CA is intact, the CBF remains relatively static irrespective of ABP changes. This allows CA measurement by assessing the transmission of changes in driving pressure (ABP or CPP) to changes in CBF.^16,17^ Flow-based neuroimaging techniques represent the gold standard for CBF measurement,^18^ but are not practical in critically ill patients and carry radiation/radionuclides exposure risk. So, alongside continuous invasive monitoring of ABP, several surrogates for CBF are used to assess CA.^19,20^ Some of the commonest surrogates used are: a) CBF velocity (CBFV) as measured by Transcranial Doppler (TCD)^17,21^ b) cerebral regional saturation of oxygen (CrSO_2_) measured by near-infrared spectroscopy (NIRS)^22^ c) brain tissue oxygenation (PbtO_2_) measured via an invasive probe as the partial pressure of oxygen in the brain tissue, as a surrogate of conductive and diffusive oxygen delivery^23^ and, d) intracranial pressure (ICP) as a surrogate of cerebral blood volume (CBV).^24,25^

Simplistically, CA can be assessed using static and dynamic methods.^26^ The static methods of CA assessment use an induced step change in ABP and study the resulting shift in CBF surrogates. Dynamic methods assess the transient behaviour of CBF surrogates resulting from a spontaneous or induced change in ABP/CPP. Given the intermittent nature and risks associated with inducing ABP changes in critically unwell patients, dynamic methods have gained popularity in critically ill population as they use the transmission of natural oscillations in ABP to the CBF surrogates.^27^ Time or frequency domain analysis of the association between the signals representing systemic and cerebral circulation, allows calculation of an autoregulatory index which provides continuous real time information about the state of CA. Many CA indices have been developed based on different signals and different calculation methods.^28^ Although the detailed discussion of the methodology and the indices is beyond the scope of this article, Table 1 summaries the most commonly studied CA indices. The CA information thus obtained can help determine optimum CPP (CPPopt)^29,30^ or ABP/MAP (ABPopt/MAPopt)^31^ targets respectively, at which the CA is most preserved or least impaired. Further, ABP or CPP at LLA^32^ and ULA^33^ can be assessed. This can help individualising treatment targets for patients in real time.

**Table 1.** Summary of the main cerebral autoregulation indices currently used in paediatric settings.

CA index	Signals	Calculation & interpretation	Proposed cut-off for impaired CA, unless specified otherwise
RoR (rate of autoregulation)	MAP & CBFV	(ΔCVR /Δ T): ΔABP- Rate of restoration of CBFV with respect to a dynamic drop in ABP (e.g., during seat to stand exercise)	NA^48^
Rate of CBFV change index (static CA)	MAP & CBFV	Percentage of change in CBFV over a unit change in ABP, induced by pharmacological means- %ΔCBFV/ΔABP	NA^142^
Static ARI (static autoregulation index)	MAP (or CPP) & CBFV	Calculated as a relative change in cerebrovascular resistance (CVR = ABP/CBFV, or CPP/CBFV) over relative change in ABP or CPP) respectively: %ΔCVR/%ΔABP, or %ΔCVR/%ΔCPP	<0.4^37–47,73,77,78,117^
Dynamic ARI (dynamic autoregulation index)	MAP & CBFV	Comparison of the CBFV response to a transient step change in ABP with those predicted from a parametric model at 10 different levels of CA. The step ABP change is induced by rapid deflation of thigh cuffs, however dARI could also be calculated from spontaneous waves using transfer function analysis.	0 completely impaired CA5 Intact CA9 Overreactive CA^76^
Slope of autoregulatory plateau	MAP, CBFV, PaCO_2_	Slope of CBFV to ABP from a linear multivariate model including ABP and PaCO_2_ during a provocative test (tracheal suctioning)	>0^117^
CBFV slope of transients	MAP & CBFV	The temporal pattern of CBFV transients on response to ABP transient, is classified according to clusters of autoregulatory response	NA^137^
Mx (mean index of autoregulation) Sx (systolic index of autoregulation) Dx (diastolic index of autoregulation)	CPP (or MAP) & CBFV	Person correlation coefficient between CPP and mean CBFV (Mx) (300-s window of 10-s averages). There exist several variants of the method: Mxa (using MAP instead of CPP), systolic values of ABP and CBFV (Sx), diastolic values of ABP and CBFV (Dx)	Mx: >0.45^17,124^
PRx (pressure reactivity index)	MAP & ICP	Moving Pearson correlation coefficient between invasive MAP and ICP (300-s window of 10-s averages)	>0.3^52–54^>0.2^11,51^
LAx (low frequency autoregulation index)	MAP & ICP	Low frequency version of PRx, based on minute-by-minute data, the period of calculations is not standardized and can range from 5 min to 2 hours.	>0.2^12,60–62^
COx (or TOx) Cerebral oximetry index orTotal oxygenation reactivity index	MAP or CPP & CrSO_2_	Pearson correlation between MAP and cerebral oxygen saturation	>0.5^112–115,126–128,133,138,141^≥0.4^85,140^>0.3^66,115^>0^170^NA^99,102,107,116,139^
MAP-CrSO_2_ repeated measures correlation coefficient	MAP & CrSO_2_	Repeated measures correlation between epochs of MAP and cerebral oxygen saturation over a period.	NA^74^
MAP-cFTOE correlation coefficient	MAP & cFTOE	Pearson or Spearman correlation between cFTOE and ABP, calculated over pre-defined epochs.	≤−0.3^121^<−0.5^115^NA^116^
HVx (or THx) Haemoglobin volume reactivity index orTotal haemoglobin reactivity index	MAP or CPP & tHb	Pearson correlation between 30 consecutive 10-s means of ABP and tHb	>0.3^87^
DCSx Diffusion correlation spectroscopy blood flow reactivity index	MAP & DCS based Blood Flow Index BFI	Pearson correlation coefficient between MAP and microvasculature blood flow, BFI, values, sampled every 7 seconds, from a period of 30–60 min	NA^96^
TOHRxTotal oxygenation heart rate index	Heart rate & cerebral oxygenation	Moving correlation coefficient between cerebral oxygenation and HR (300-s window of 10-s averages)	NA^110,111,132,152–154^
Wavelet IndiceswPRxwCOx wHVx	NIRS signals or ICP & ABP	Wavelet analysis based PRx, and HVx, calculated as a cosine of the phase shift between slow waves in NIRS parameters or ICP and ABP	wPRx: >0.24^59^wCOx: >0.26^94^wHVx: >0.19^59^; NA^171^
MAP- CrSO_2_ COH	MAP & CrSO_2_	Spectral coherence of slow waves analysis	≥0.5^103,129,135^ ≥0.45 (low frequency)^136^≥0.47 (very low frequency)^136^>0.3^144^NA^99,102,107,139,143,147,159^
MAP-HbD COH	MAP & cerebral intravascular oxygenation (HbD = HbO_2_ – HHb)	Spectral coherence of slow waves analysis	>0.5^105^>0.384^151^NA^129,160,163,175,177^
MAP-CrSO_2_ TF gain	MAP & CrSO_2_	Spectral transfer function gain (TF) in slow wave frequencies (0.002–0.02)	NA^102,104,134,139^Mean Gain +2SD^144^
MAP-HbD TF gain	MAP & HbD	Spectral TF gain analysis	NA^130^
HVP	MAP & cerebral tHb	Cosine-transformed phase shift at maximal coherence between tHb and ABP	>0.34^162^, NA^161,163^
Bivariate phase rectified signal averaging (BPRSA)	MAP/HR & CrSO_2_	Bivariate autoregressive modelling based spectral estimation	NA^131^
Bivariate autoregressive spectral coherence (BiAR-COH)	MAP & CrSO_2_	Bivariate autoregressive modelling based spectral estimation	>0.57^100^
Partial directed coherence (PDC)	MAP & CrSO_2_	Bivariate autoregressive modelling based spectral estimation	>0.55^101^

ABP: arterial blood pressure; ARI: autoregulatory index; CA: cerebral autoregulation; CBFV: cerebral blood flow velocity; cFTOE: cerebral fractional oxygen extraction; COx: cerebral oxygenation index; CPP: cerebral perfusion pressure; CrSO_2_: cerebral tissue oxygenation index; CVR: cerebro-vascular resistance; HbO_2_: oxygenated haemoglobin; dARI: dynamic ARI; HbD: haemoglobin difference; HHb: deoxygenated haemoglobin; HR: heart rate; HVx: haemoglobin volume reactivity index; HVP: haemoglobin volume phase index; ICP: intracranial pressure; LAx: low frequency autoregulation index; Mx: mean flow index; MAP: mean arterial blood pressure; NA: not available; PaCO_2_: partial pressure of arterial carbon dioxide; PRx: pressure reactivity index; rTHb: relative total haemoglobin; sARI: static ARI; T: time; TF: transfer function; tHb: total haemoglobin; THx: total haemoglobin reactivity index; TOHRx: total oxygenation heart rate index; wCOx: wavelet COx; wHVx: wavelet HVx; wPRx: wavelet PRx.

The available scientific data on CA assessments in the paediatric population is limited and heterogeneous. This scoping review aims to analyse the published literature on CA assessment in the paediatric and neonatal settings with a goal to improve understanding of CA derangement burden and understand gaps in knowledge for clinical implementation of CA assessment in this population. In particular we investigate conditions where CA has been assessed, methods and CA indices which are most robust in paediatric CA monitoring and the relationship between disturbed CA and outcome in this population.

## Methods

The first and the senior authors decided on the search string for the scoping review which was subsequently adapted to different databases searched: ((“cerebral autoregulation” OR “cerebral regulation” OR “cerebrovascular regulation” OR “cerebrovascular autoregulation” OR “pressure reactivity” OR “cerebrovascular reactivity”) AND (“Infant*” OR “Child*” OR “neonat*” OR “paediatric*”)). Three bibliographic databases were searched for studies published within 31st December 2022, Medline (http://www.ncbi.nlm.nih.gov/pubmed/), Scopus (https://www.scopus.com) and Web of Science (https://www.webofscience.com/wos/woscc/basic-search). For each database search, filters to exclude book chapters, reviews and meta-analyses, study protocols, editorials, commentaries, consensus statements or expert opinion articles, meeting abstracts and studies performed on animals, adults or published in languages other than English, were applied. No protocol registration was performed for this review.

The first authors checked the obtained search records for duplicates initially. As a second step, two reviewing authors screened and evaluated titles and abstracts of the records for eligibility (i.e., clinical trials, observational studies, interventional studies on paediatric/neonatal CA) and exclusion criteria (i.e., book chapters, reviews, meta-analyses, study protocols, editorials, commentaries, consensus statements, expert opinion articles, meeting abstracts, studies performed on animals, adults or published in languages other than English). Third, full-text screening was conducted independently by two authors. Studies in which CA was not assessed, or with mixed adult and paediatric cohorts without separate paediatric results, were excluded during the full-text eligibility screening. Any discrepancy was first discussed between the first authors, and in case consensus could not be reached, two additional co-authors were involved to reach consensus.

The data were extracted manually by the reviewers. The elements extracted included type of methodology or metric used for monitoring of CA, details about the study population (number of participants and type of pathology), study type (with specific interventions in case of interventional studies), key findings relevant for this review, in particular any threshold on the metric of CA that was associated with any outcome in the study. Being a scoping review, the quality of the papers included was not individually assessed as in systematic reviews; however, based on the study characteristics and methodology, a global quality assessment of the examined literature has been performed and is discussed in the discussion section.

The scoping review results are presented in four sections based on the populations or conditions addressed by the included records.

## Results

**Figure 2.**
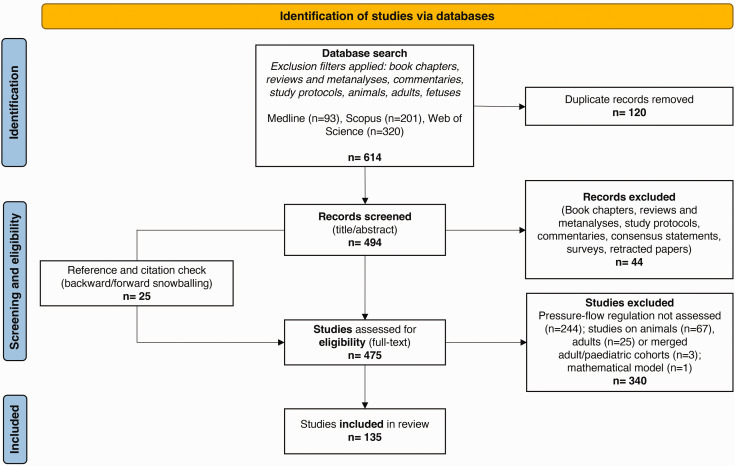
PRISMA diagram. It maps out the different research phases and the papers in the review process.

As shown in the PRISMA flow diagram (Figure 2), out of 494 screened, 135 studies are included in this review. A detailed presentation of the records collected, inclusive of the aspects considered for data extraction, study descriptions and key findings are available as supplemental material (Appendix 1). We also discuss details of the methods and CA indices adopted in the studies included in this scoping review. The list of these indices, together with their description, is available in Table 1 and summarised briefly in the paragraph “Summary of methods used for CA assessment”.

In the section “Summary of studies” we present results of the literature search with some context background at the beginning of each section to help the reader.

## Summary of methods used for CA assessment

We have identified mainly 24 methods used for CA assessment in paediatric age group (Table 1), the majority being CBFV or NIRS based. While 11/24 methods use time-domain metrics (64 studies), 12/24 use frequency domain metrics (67 studies) and both metrics together are used in 4 studies. Majority studies use dynamic assessment of CA (86%) corresponding to 118 studies. In PTBI, the most common methods were ICP based (PRx and LAx, 15 studies) and CBFV based (sARI, 11 studies). In paediatric cardiac surgery the most common methods were NIRS based (11 of 12 studies). NIRS based methods were also used in 2/4 post cardiac arrest encephalopathy and 2/3 paediatric stroke studies. In the neonatal population, the vast majority of CA assessment was performed using NIRS (82%, 64 studies) followed by CBFV (11%, 9 studies). In healthy children 3 studies were CBFV based, and 2 studies were NIRS based.

## Summary of studies

Based upon the patient groups where CA was assessed, included studies are classified into the following five sections: a) paediatric acute brain injury, b) other paediatric conditions c) healthy children d) paediatric cardiac surgery and extra corporeal membrane oxygenation (ECMO), and e) neonatal CA. The full list of the studies with detailed information is provided in Appendix 1. We present results of the literature search with some context background at the beginning of each section to help the reader.

### Acute paediatric brain injury (36 papers)

#### Paediatric traumatic brain injury (TBI)

In the paediatric population, CA has been most widely studied in TBI. CA is crucial for maintaining brain homeostasis by providing uninterrupted oxygen and substrate supply and is frequently deranged in TBI contributing to secondary brain injury in children.^34^ We found 29 paediatric TBI studies that tested CA using different indices (Appendix 1).

TCD based CA assessments were the first to appear given their non-invasive nature. Autoregulatory Index (ARI) was the first such CA index reported in TBI and is defined as a ratio of relative change in cerebrovascular resistance (CVR) to an induced change in ABP (or CPP). To distinguish static ARI from dynamic index of autoregulation with the same acronym,^35,36^ we use sARI for static and dARI for dynamic ARI respectively. In paediatric TBI, sARI has shown correlation with outcome^37–40^ and it can also assist in clinical decision making.^41–47^ Using sARI, Figaji et al. demonstrated that autoregulation differed among individuals and the state of CA influenced the response of ICP and PbtO_2_ to changes in ABP in severe TBI (sTBI).^41^ CA impairment is also frequent (48.4% patients, sARI < 0.4) in mild TBI (mTBI) and can involve both the ipsilateral and contralateral hemispheres.^42,43^ Independent of the TBI severity, younger age (<4 years) is shown to be a risk factor for impaired CA as measured with sARI.^44^ Vavilala et al. demonstrated diffuse, hemispheric differences in CA measured with sARI in focal TBI, revealing that CA was often impaired in the apparent “unaffected” hemisphere and that normal neuroimaging was not always associated with intact CA.^45^ However, sARI involves inducing step increase in ABP, which could be potentially harmful in brain-injured children, and it does not allow continuous CA assessment. So, after the early enthusiasm, sARI use is mainly restricted to mTBI more recently. The other TCD based indices are Rate of Regulation (RoR) with a sit-to-stand protocol, feasible only in awake children with concussion,^48^ and TCD-derived Pulsatility Index (PI),^49^ the utility of which for CA assessment has been questioned.^50^

As children with sTBI are managed with invasive ICP and ABP monitoring, the relationship of these two signals has been used for continuous CA assessment without requiring ABP challenge. Increasing evidence supports the methodology; amongst several available indices, PRx (pressure reactivity index) is the most studied so far. It is calculated as Pearson’s moving correlation coefficient between the slow wave fluctuations in ABP and ICP, reflecting an intact CA with negative, and impaired CA with positive correlation, respectively. LAx (low frequency autoregulation index), low frequency version of PRx, is also commonly used (Table 1). In our review, 15 studies measured CA using PRx or LAx and majority showed a significant association between CA and outcome.^11,12,15,51–62^ Brady et al. found that children who died had significantly higher PRx and that PRx could be CPP-dependent in TBI.^54^ Lewis et al. observed that PRx had a prognostic value and could identify CPP targets.^52^ Young et al. showed that PRx and deviation of CPP from derived CPPopt correlated with patient outcome.^51^ Abecasis et al. compared PRx with Mx (mean flow velocity index using CBFV measured by TCD) and COx (cerebral oximetry index using NIRS) and found PRx as the most robust index for CA assessment and, both PRx and COx useful for CPPopt assessment.^55^ Apart from the average value of the CA index, the length of time spent with impaired CA (percentage of monitoring time with PRx > 0 approximately higher than 50%, or time spent with LAx > 0.2), has also shown association with worse outcomes in sTBI.^11,12^ Newly refined PRx methodology like wPRx (wavelet PRx) and other model-based indices to assess CA were recently used by Appavu et al. in a retrospective single centre cohort of sTBI patients showing improved outcomes with CPP above the PRx derived LLA.^15^ Appavu et al. also argued that elevated value of PRx and wPRx denoting impaired CA are predictive of post-traumatic epilepsy.^57^ Two studies investigated effects of hyperosmolar therapy on PRx; one study showed a restoration of CA with hypertonic saline in the favorable outcome group^58^ and the second study showed a decrease in PRx and wPRx.^59^ Young et al showed an association of raised systemic glucose with impaired CA as measured by PRx after sTBI.^56^ A reduced ability to tolerate ICP insults ensuing from impaired CA were observed using LAx.^12,60^ Guiza et al^61^ used LAx and derived CPPopt with a composite, DATACAR algorithm which showed significant predictive power of deviation from CPPopt. Using the same approach, in a pilot analysis, Lo et al. reported that CPPopt varied with time during patients’ stay in PICU and that among patients with good outcome, the time spent with CPP within CPPopt was significantly higher.^62^

NIRS-derived indices of CA,^20^ although attractive for their non-invasive nature, have shown conflicting results in TBI and hence are not widely used in this context.^63^ Except for the LAx based studies above, majority of the published literature of CA assessment in paediatric TBI come from single centre studies. There are two ongoing prospective multi-centre studies, using PRx^64^ and LAx^62^ respectively, which will give much needed data on the utility of CA assessment through model-based indices in this patient population. The technique holds promise in its ability to help individualise treatment targets based on the state of CA in real time by calculating CPPopt, LLA and ULA.

#### Post-cardiac arrest encephalopathy

Outcomes from paediatric cardiac arrest are poor with a high risk of long-term neurologic disability in those who survive. Only a few studies have investigated the role of CA in this patient group, mainly with NIRS derived CA indices, namely, COx or HVx (Haemoglobin volume index).^20^

A study in 36 post arrest children using HVx showed that the amount of time spent below the HVx derived ABPopt (greater Area Under the Curve below ABPopt) was associated with unfavourable outcomes.^65^ This finding was confirmed by another recent study, which used COx to define ABPopt targets and found worse outcomes in children with larger deviations of ABP below the calculated ABPopt and with more time (38% of monitored time) spent with ABP below ABPopt.^66^ Interestingly, Zipfel et al monitored CA with PRx in 19 children and showed that impaired CA within 72 h after resuscitation is associated with unfavourable outcome.^67^ Only a small paediatric case-series evaluated CA intermittently by using TCD after a global hypoxic-ischaemic event and showed a near-normal CBFV and an intact CA in children with favourable neurologic outcome.^68^ It however remains unknown whether prospectively targeting ABPopt in these patients could improve neurological outcomes.

#### Paediatric stroke

Stroke is rare in children, and we found only three published studies assessing CA in this group. Moyamoya disease carries a high risk of ischaemia and requires surgical revascularization; Lee and colleagues used NIRS based CA indices in this population and were able to identify intraoperative and postoperative ABPopt (86% of patients) and intraoperative LLA (43%) in their first pilot study.^69^ The authors also carried out a follow-up study, concluding that poorer CA during surgery was associated with postoperative transient ischemic attacks in children with bilateral vasculopathy.^70^ Appavu et al reported on CA in children with ruptured cerebral arterio-venous malformation (AVM) and observed that deranged CA, dysautonomia and a longer time spent below the LLA were associated with poorer outcomes and acquired epilepsy at 12-month follow-up.^71^

### Other paediatric conditions (4 papers)

CA has not been studied much in other neurological conditions in children. There are single studies each reporting CA derangements in, a) survivors of paediatric brain tumours,^72^ b) children with diabetic ketoacidosis where deranged CA seems to be common,^73^ c) sevoflurane anaesthesia, where CBF seems to be “pressure dependent” indicating a poor efficiency of CA as assessed by CrSO_2_,^74^ and d) children with congenital central hypoventilation syndrome where CA appears intact as measured with COx, unless ABP is lower than LLA.^75^

## Healthy children (5 papers)

CA has been assessed non-invasively in healthy children. Vavilala observed that dARI (based on the CBFV in the middle cerebral artery, MCA) is physiologically lower in adolescent (12–17 years) than in adults.^76^ Though no gender differences were found in CA, higher FV was seen in girls.^77,78^ NIRS-based oxygenated haemoglobin was tested in healthy children during postural changes which confirmed that NIRS assessment of CA was reliable to detect children with abnormal cerebrovascular response.^79^ Wagner et al. found a significant correlation between cerebral Hb signals and direct CBF measures after a bolus of phenylephrine (PE) and concluded that non-invasive NIRS and single dose PE can reliably determine dynamic CA.^80^

### Paediatric cardiac surgery and ECMO (12 papers)

#### Cardiac surgery

Though mortality in infants with congenital heart disease (CHD) has decreased over the last decades, neurological morbidity in the survivors is concerning.^81,82^ All of the literature available regarding paediatric cardiac surgery considers the intraoperative and the postoperative period, this makes it difficult to differentiate between CA impairment due to the CHD itself or due to other interventions.

**Figure 3.**
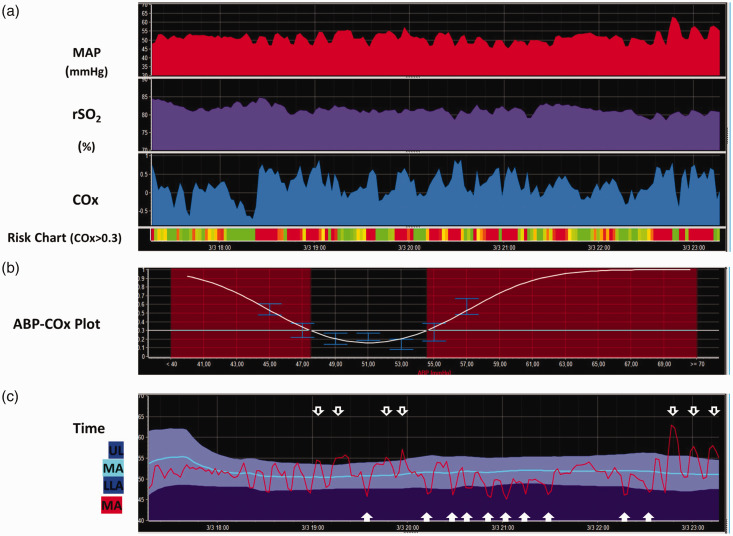
Autoregulation monitoring in a neonate post-cardiac surgery. Signals from invasive arterial blood pressure and cerebral NIRS with INVOS^TM^ were collected using ICM+ software (University of Cambridge, UK) for continuous Cox calculation (Figure 1(a)). A multi-window weighted algorithm based on 8-hour epochs was used to obtain the autoregulation U-shaped curve (Figure 1(b)). Figure 1(c) shows the time trends of COx-derived optimal MAP (mean arterial blood pressure), LLA (lower limit of autoregulation) and ULA (upper limit of autoregulation). The percentage of time spent with COx > 0.3 and MAP < LLA or MAP > ULA were provided as COx-derived metrics aimed to quantify the cumulative burden of the episodes with impaired CA.

Although the exact causes of neurological injury in CHD still need to be defined, the physiological changes caused by CHD and the surgical procedures needed to treat them carry significant risks to the developing brain with a cumulative effect.^83^ Monitoring CA during and following cardiac surgery can be crucial in understanding and preventing brain injury. NIRS based COx and HVx indices are particularly suited as they are non-invasive and technically easy to use^84^ (Figure 3). Several factors unique to CHD patients must be considered when monitoring the paediatric brain during cardiopulmonary bypass (CPB) such as: the type of lesion, use of hypothermia or deep hypothermic circulatory arrest (DHCA), low flow CPB, selective cerebral perfusion, use of ECMO and Ventricular Assist Devices (VAD).

Brady et al. first introduced COx and COx derived LLA in paediatric cardiac surgery in 2010; they enrolled 54 children undergoing CPB and outlined a pressure auto-regulation curve for each patient choosing a COx cut-off value of 0.4.^85^ They showed that COx remained elevated during CPB and was associated with hypotension. However, cyanotic children were not included and hypothermia or DHCA were not addressed in this study. Smith et al. examined the impact of temperature in neonates undergoing CPB and found that hypothermia and hypotension were associated with more positive HVx values, indicating impaired CA.^86^ Easley et al. found a rise in HVx with drop in MAP <40 mmHg, which suggested impaired CA; the authors identified MAPopt (48 ± 11 mHg) in all infants, whereas the LLA was identified in 82% of the patients.^87^ Both cyanotic and acyanotic CHD were included in this study and no difference observed in CA metrics between the two subgroups. The same group measured HVx in 16 patients undergoing cavopulmonary anastomosis and concluded that elevated MAP and pulmonary arterial pressure were associated with a positive HVx.^88^

Several authors have studied CA in neonates undergoing CPB. Votava-Smith et al. found impaired CA in all 24 term neonates studied at some time point during the preoperative period.^89^ Bassan et al. found disturbed CA in 51% of patients on a fluctuating basis, with abnormalities seen about 15% of the time studied after cardiac surgery in infants <7 months of age.^90^ Zipfel and colleagues compared COx and HVx in 36 infants after CPB and were able to identify MAPopt in 90.8% of these infants; MAP was more often below than above MAPopt and the LLA was higher than age-appropriate recommendations in ≥50% of patients.^91^

#### CA and ECMO

CA data in paediatric ECMO are limited. In a recent multicentric study analysing 29 children on ECMO, Joram et al. were able to obtain COx values for all patients and MAPopt, LLA, and ULA for 90% of patients.^92^ Children who developed an acute neurological event had higher COx and spent more time above the autoregulatory threshold of COx > 0.3; in addition, they also spent more time below LLA and above ULA.^92^ The same authors retrospectively analysed the relationship between PaCO_2_ and CA in 30 ECMO children and concluded that hypercapnia seems globally protective in normotensive or hypertensive conditions, while hypotension may disturb CA as it increases LLA.^93^

Tian et al. used wavelet transform coherence and phase shift between ABP and CrSO_2_ slow waves to describe the differences in cerebral hemodynamic profiles in children with no brain injury (n = 23) as opposed to children with acquired ischemic (n = 16) and haemorrhagic (n = 8) brain injury after ECMO. Patients with ischaemic brain injury had significantly elevated index values at the lower end of the ABP range.^94^ However, the index used an unusually ultra-low frequency of 0.0005–0.002 Hz (as opposed to the standard 0.02–0.2) and requires cautious interpretation.

Ortega et al. showed a significant relationship between pro-inflammatory response, loss of CA and acquired brain injury in 20 ECMO patients (0–14 years).^95^ Recently, Busch et al introduced a novel approach with a non-invasive hybrid optical based method for cerebral monitoring during ECMO, which uses diffuse correlation spectroscopy and frequency-domain diffuse optical spectroscopy to assess CA with promising early results.^96^

### Neonatal CA (78 papers)

Neonatal CA has been studied using mainly non-invasive techniques and several different analytical methods.^97–103^ We will present the current literature on CA in preterm and term infants separately.

#### CA in preterm infants

Most of the CA studies in preterm infants’ use non-invasive techniques (NIRS = 46, TCD = 11, diffuse correlation spectroscopy, DCS = 1); only 2 studies used Xe-133 clearance. Preterm infants show impaired CA, which worsens with decreasing gestational age (GA).^104,105^ Particularly, diastolic blood flow velocity derived CA dysregulation seems to persist across different gestations.^106^ However, it is unclear whether impaired CA is associated with poor neurodevelopmental outcomes due to lack of robust evidence.^107^ Targeting MAP at CA guided MAPopt may improve the long-term outcome given that the autoregulating range may be very narrow in preterm infants.^108^ Although a breakpoint in CBF-MAP relationship at approximately 30 mmHg has been suggested,^109^ current evidence indicates that the range varies between individuals and significant deviations below or above this range are associated with increased mortality and neurological sequelae.^110,111^

Studies report impaired CA in common conditions associated with prematurity like, a) intrauterine growth-restriction (for up to 5 postnatal days),^112–114^ b) respiratory distress^115,116^ (although the influence of PaCO_2_ on autoregulatory response during mechanical ventilation remains inconclusive),^117–119^ c) necrotizing enterocolitis and spontaneous intestinal perforation,^120,121^ d) persistent patent ductus arteriosus (PDA) within first 6–12 hours after ligation^122^ (greater impact on CA with posterolateral thoracotomy compared with sternotomy^123^) though the size of the PDA was not found to significantly affect CA,^124^ and e) intraventricular haemorrhage (IVH) with early Xe-133 clearance data demonstrating absent CA in infants who developed IVH,^125^ which was further corroborated by studies using different NIRS based indices showing higher time percentage with impaired CA in association with IVH, as assessed by COx,^126–128^ ABP-oxygenated haemoglobin (HbD) coherence,^129,130^ and CrSO_2_.^100,101,131^ There is contradictory evidence with some studies showing no significant difference in COx between IVH cases and controls,^132,133^ whilst delayed cord clamping in extremely preterm infants has shown association with better CA and reduction in IVH rates.^134^ Maternal chorioamnionitis has not shown association with abnormal postnatal CA.^135–137^

Various drugs used both pre- and postnatally can influence CA and have been a focus of many studies. Tocolytic indomethacin and magnesium sulphate (administered 24 hours prior to delivery) had no effect on COx,^112,138^ unlike maternal labetalol which was associated with a higher transfer function (TF) gain between MAP and CrSO_2_.^139^ The impact of dopamine on CA is inconclusive so far: a) treated neonates had longer periods with COx > 0.5 (but the concomitant hypotension could be a potential confounder),^140,141^ b) increased CrSO_2_-MAP coherence in a small cohort of treated infants compared to controls with similar MAP,^135^ c) increase in pressure-passive CBF with dopamine use in hypotensive infants during the first 24 h,^142^ and d) Hypotension in Preterm Infants trial, a randomized controlled trial of dopamine vs placebo in hypotensive infants, did not demonstrate significant CA differences in the two groups, though the low number of treated infants may have underpowered the analysis.^143^ Propofol administration maintained intact CA in most neonates despite drug-related hypotension.^144^ Pancuronium-mediated paralysis in mechanically ventilated preterm infants was shown to increase CBF dependency on MAP.^145^ In a trial comparing atropine-propofol vs. atropine-atracurium-sufentanyl for intubation sedation, no between-group difference was observed in MAP-cFTOE (cerebral fractional oxygen extraction) correlation.^146^ Caffeine load was found to reduce COx in preterm neonates.^147^ As for surfactant administration, less invasive techniques had more preserved CA than intubation.^148^

Invasive ABP monitoring can be challenging in preterm infants. Heart rate (HR), as a determinant of cardiac output, has been used as a surrogate for ABP and alternative CA indices using HR have been proposed.^111,132,149–154^ As an example, the moving correlation coefficient between cerebral oxygenation and HR, TOHRx (tissue oxygenation heart rate reactivity index), has been suggested as marker of CA in preterm infants.^132,133,152^ Positive TOHRx values suggest impaired CA and have been associated with higher CRIB-II, which predicts mortality amongst low birth weight infants.^111,153–155^ However, the high prevalence of baroreflex dysfunction in preterm infants has raised concerns on the reliability of the correlation between HR-HbD coherence for CA assessments and we will not discuss these indices in detail.^152^

#### CA in term neonates

In term infants, CA was studied using NIRS (n = 19), TCD (n = 2), Xe-133 clearance (n = 1), and thermal diffusion flowmetry (TDF) (n = 1). Although CA maturation is almost complete in term neonates, significant disturbances can occur under pathological circumstances, such as hypoxic-ischaemic encephalopathy (HIE). Persistent cerebrovascular vasodilation in HIE may disrupt CA.^156^ Using Xe-133 clearance, Pryds et al. first described a transient cerebral vasoparalysis with abolished vascular responses to ABP fluctuations in neonates with HIE.^157^ Similar passivity has been seen with TCD.^158^ Various NIRS derived parameters report worse outcome in HIE with impaired CA as predicted by: a) wavelet coherence between MAP and CrSO_2_,^159^ b) spectral coherence between MAP and NIRS signals,^160^ c) haemoglobin volume phase index (HVP).^161,162^

The accuracy of CA indices in predicting HIE-related death or brain injury can be influenced by temperature and postnatal age.^163^ Therapeutic hypothermia (TH) is the gold-standard treatment to reduce neurological sequelae in moderate to severe HIE cases.^164^ Increased incidence of MRI abnormalities and psychomotor impairment at 21–32 months was observed with prolonged deviations below MAPopt during TH or rewarming^165–170^ and a lower incidence of brain injury seen if MAP > MAPopt during TH.^171^ Deviations above or below MAPopt in HIE infants have also shown association with extra-cerebral outcomes.^172,173^ Gilmore et al. showed that temperature fluctuations during TH were associated with a MAP > MAPopt shift.^174^ Limited data on mild HIE show no difference in CA across different brain regions or between areas with neurological lesions and intact ones.^175^

In infants who underwent CSF diversion for congenital hydrocephalus, an absent CA as measured invasively using TDF, was associated with increased rates of death and severe developmental delay.^176^ In the study by Govindan et al., ventilator related CBF fluctuations, as assessed by spectral power of total haemoglobin at the ventilator frequency, were associated with poorer CA and higher risk of brain injury.^177^

## Discussion

It is well acknowledged that CA changes over time and between individuals; there are age and physiology-related differences in CBF, ABP, and brain metabolism.^178^ Our literature search highlights areas where CA has been studied and explores possible clinical implications as well as limitations in paediatric population. The most important limitations this review highlights are the variability of methods used to calculate CA indices, as evidenced in Table 1, and the heterogeneity of adopted cut-off thresholds, some of which are empirically established pending validation in larger studies. Recently, Liu et al. were able to identify the thresholds of intact and impaired CA for three indices, Mx, COx and HVx in a cohort of 59 patients undergoing CPB. They derived ‘Lassen’ curve by plotting TCD FV values against ABP and identified LLA for each patient and compared it against the CA indices.^179^ Although elegant, the findings require confirmation in a larger cohort. In general, the precise thresholds for most CA indices remain somewhat elusive, which hinders comparability of the available evidence and application of CA monitoring in routine clinical practice. Another potential limitation is that the group of paediatric TBI patients may be over-represented among patients’ categories, due to the clinical need of invasive ICP monitoring and thus making CA monitoring available via ABP and ICP modalities. This could introduce a selection bias in CA monitoring research. More importantly, there is no gold standard in CA assessment, particularly one applicable to dynamic, continuous measurements that can be used to benchmark the accuracy of the proposed methods. As each method is associated with a multitude of assumptions, some wilder than the others, a high degree of caution must be exercised when interpreting results reported in the papers, which often are over optimistic or categorical.

Thus said, overall, there seem to be enough good quality evidence in the literature to support the importance of CA assessment and continuous monitoring for management of paediatric patients across ages with several conditions and pathologies. Most literature on infants and children is aimed at establishing an association between CA monitoring and outcomes in the context of clinically overt brain injury. Current evidence in paediatric acute brain injury, although limited, demonstrates that the amount of time spent with deranged CA and the magnitude of CA derangement have a negative impact on outcome, both in terms of mortality and disability. Therefore, considering deranged CA as a digital biomarker for acute brain injury severity and outcome prognostication could already be suggested for clinical implementation.

Also, at CPP or ABP values beyond the upper or lower functional range of CA, autoregulatory mechanisms are no longer effective and CBF becomes pressure-passive, potentially leading to secondary brain injury and worse outcomes. The relationship between the degree of ABP deviation from optimal values and outcome needs to be explored prospectively.

The safety and feasibility of a CA-targeted CPP management has been recently demonstrated in adult TBI patients.^29^ The current recommendation from paediatric TBI guidelines to maintain CPP at a minimum of 40 mmHg is comprehensively weak.^180^ It follows that continuous CA monitoring and prospective studies can be instrumental to help identify individualized treatment and CPP-ABP targets based on the state of CA in this patient population.^181–183^

For patients undergoing cardiac procedures, targeting adequate ABP to preserve brain and end-organ perfusion is fundamental. As described above, there is no agreement on the definition of an adequate ABP. This is challenging, since maintaining an adequate ABP is not limited to the perioperative period but extends to the intensive care period and beyond. As the timing at which impaired CA begins and its relationship with the onset of cerebral injury is still not known, it further complicates implementation of CA monitoring. In an adult study, Joshi et al. demonstrated that the limits of autoregulation may vary greatly and unpredictably between patients and that LLA cannot be predicted based on demographic and disease-specific information.^184^ If this concept is true in adults, it is even more relevant in children with CHD who have complex circulations and age-related changes from preterm to young adulthood. Furthermore, the role of changes in cardiac output in children undergoing complex cardiac procedures in changing CBF or CA mechanisms is as yet unknown.^185^ Moreover, mechanical ventilation or lung diseases may be associated with significant PaCO_2_ fluctuations, with possible effects on the LLA and ULA.^186,187^ Seizures can interfere with cerebral haemodynamic in children and infants. For this reason, a continuous assessment of CA before, during and post cardiac surgery, in the context of a multi-modal-monitoring, could be suggested to better understand brain haemodynamic and its relationship with other physiological variables, with the final goal to prevent acute neurologic complications in this peculiar clinical setting.

A significant proportion of neonatal studies aim to shed light on CA mechanisms in different physiological or pathological settings.^188^ An important characteristic of this population, especially if born preterm, is that multiple pathophysiological conditions associated with altered CA may coexist, adding up to the risk of brain injury. As in the paediatric population, the role of CA monitoring to define optimal ABP targets may represent an adjunctive neuroprotective strategy not only in HIE infants but also in preterm neonates, to optimize their autoregulatory capacity and to prevent the burden of neonatal brain injury and the related long-term consequences.

The overall quality of the literature examined was also globally reviewed, with particular reference to the study design (e.g., sample size, methods used for CA assessments, availability of a control group etc; for study details, see Appendix 1). A critical point of the reviewed literature was the observational nature of most of the studies examined, with lack of control groups based on healthy children/neonates. Moreover, more than half of these studies were based on small samples (n < 40), which may pose a potential limitation to the generalizability of the study results. Finally, the methodological variability for CA assessment, which has been previously discussed, represents a possible bias and hinders the feasibility of result comparisons between different studies.

## Conclusion

The findings from this literature scoping review highlight that various attempts have been made of monitoring of CA in both paediatric and neonatal populations, however there is lack of standardization of the methodology for CA assessment, including cut-off values for indices and criteria for impaired CA. This hinders the translation of CA monitoring into routine paediatric and neonatal intensive care practice. Moreover, current literature in children and neonates is mostly based on small observational studies with significant physiological and pathophysiological heterogeneity that hampers the generalizability of the obtained results. Nevertheless, the impairment of CA seems to be associated with worse neurological outcomes and CA monitoring in the context of a more extended multimodal monitoring has the potential to individualize patient management. Hence, implementing CA monitoring would represent an important goal to implement neuroprotection in these populations. Adequately powered multicentre outcome studies using standardised methodology are required to overcome this limitation.

## Supplemental Material

sj-pdf-1-jcb-10.1177_0271678X241261944 - Supplemental material for Cerebral autoregulation in paediatric and neonatal intensive care: A scoping review Supplemental material, sj-pdf-1-jcb-10.1177_0271678X241261944 for Cerebral autoregulation in paediatric and neonatal intensive care: A scoping review by Marta Fedriga, Silvia Martini, Francesca G Iodice, Cristine Sortica da Costa, Stefano Pezzato, Andrea Moscatelli, Erta Beqiri, Marek Czosnyka, Peter Smielewski and Shruti Agrawal in Journal of Cerebral Blood Flow & Metabolism
