# Supplementary material for: Cerebral autoregulation in paediatric and neonatal intensive care: A scoping review
Source: J Cereb Blood Flow Metab. 2024 Jun 13:0271678X241261944. Online ahead of print. doi: 10.1177/0271678X241261944 (PMC11542144; doi:10.1177/0271678X241261944)
Supplement: sj-pdf-1-jcb-10.1177_0271678X241261944 - Supplemental material for Cerebral autoregulation in paediatric and neonatal intensive care: A scoping review [file sj-pdf-1-jcb-10.1177_0271678X241261944.pdf]

Appendix 1

Summary of the papers included in the results

PAEDIATRIC TRAUMATIC BRAIN INJURY (29)

Authors	***CA Monitoring***	***Cut-off***	***Patient population***	***Results***
Abecasis et al. (2021)[55]	PRx, Mx, COx	PRx 0.3	12 children, sTBI	PRx is the most robust index to access cerebrovascular reactivity in children with TBI and has prognostic value. Optimal CPP calculation is feasible with PRx and COx.
Adelson et al (1998)[63]	HbD-HbO_2-Tot Hb /ICP & ABP	NS	10 children, sTBI	NIRS reliably detects changes in cerebral hemodynamics.
Appavu et al. (2021)[15]	PRx, PAx, WPRx	PRx, Pax 0.3	56 children, TBI	CA index (PRx, PAx) is associated with outcome (GOSE Ped).
Appavu et al (2022)[57]	PRx, WPRx	NS	72 children, moTBI/sTBI	Elevated PRx and wPRx are predictive of post traumatic epilepsy.
Brady et al. (2009)[54]	PRx	0	21 children, sTBI	PRx is associated with survival and is CPP dependent.
Chaiwat et al. (2009)[38]	sARI	0.4	38 children, sTBI	Impaired CA associated with poor outcome.
Deines et al. (2019)[49]	PI	NS	60 children, mTBI	No correlation between PI and outcome at 4-6 weeks.
Figaji et al. (2009)[41]	sARI	0.4	24 children, sTBI	CA testing may assist clinical decision making.
Flechet et al. (2018)[12]	LAx	0.2	259 pt, 117 children	Duration and intensity of deranged CA play a role in outcome.
Freeman et al. (2008)[44]	sARI	0.4	37 children GCS<13	Young age >4 years is a risk factor for impaired CA.
Guiza et al. (2015)[60]	LAx	0	261 total pt<br>99 children with TBI	ICP-time burden is associated with worse outcome and children have reduced ability to tolerate ICP insults.

Guiza et al. (2016)[61]	LAx	NS	79 children, TBI	CPP close to recommended CPP values was significantly associated with better outcome and was a significant independent predictor of better outcome.
Hockel et al. (2017)[11]	PRx	0.2	17 children, TBI	CA is correlated with outcome.<br>Time spent with deficient CA has impact on outcome.
Lele et al. (2018)[42]	sARI	0.4	31 children, mTBI	CA impairment commonly extends to the contralateral hemisphere.
Lewis et al. (2015)[52]	PRx	0.25	36 children, TBI	PRx has prognostic value and can identify CPP targets.
Lo et al. (2018)[62]	LAx<br>DATACAR	NS	99 children, TBI	There were significantly higher proportions with CPP monitoring time within CPPopt among patients with favourable outcome (GOS 4 and 5).
Moir et al. (2018)[48]	RoR (sit-to-stand protocol)	NS	19 concussed, 18 controls	Impaired CA after concussion, which in some case improves along with clinical symptoms.
Nagel et al. (2016)[53]	PRx	NS	10 Infant/children, sTBI	PRx correlates with 6 months outcome.
Thamjamrassri et al. (2022)[40]	sARI	0.4	24 children, TBI	The co-occurrence of hypotension and cerebral autoregulation may be a sufficiency condition needed to affect TBI outcomes.
Tontisirin et al. (2007)[47]	sARI	0.4	9 children, sTBI	CA often changes during the first 10 days from trauma.
Vavilala et al. (2004)[37]	sARI	0.4	36 children, sTBI	The incidence of impaired CA was greatest in sTBI which was associated with poor outcome.
Vavilala et al. (2006)[39]	sARI	0.4	28 children, sTBI	Impaired CA (<0.4) associated with poor 6 months outcome (GOS<4).
Vavilala et al. (2007)[46]	sARI	0.4	15 children, sTBI	CA is impaired in TBI in most cases. Inflicted TBI has worse outcome compared with non-inflicted TBI.
Vavilala et al. (2008)[45]	sARI	0.4	42 children, TBI	Hemispheric difference in CA.
Vavilala et al. (2018)[43]	sARI	0.4	6 children, mTBI	Impaired CA occurs in mild TBI.
Wellard et al. (2021)[59]	PRx, wPRx, PAx	NS	30 children,	Reduction of PRx after a bolus of hyperosmolar therapy.
Young et al. (2016)[51]	PRx	0.2	12 Children, TBI	PRx and deviation of CPP from CPPopt correlate with patient outcome.
Young et al. (2017)[56]	PRx	NS	44 children, sTBI	Increase in glucose is associated with impaired CA.

Zipfel et al. (2021)[58]	PRx	NS	28 children, sTBI	Hypertonic saline administration restored disturbed CA only in the favourable outcome group.

PEDIATRIC POST CARDIAC ARREST ENCEPHALOPATHY (4)

Authors	***CA Monitoring***	***Cut-off***	***Patient population***	***Results***
Kirschen et al (2021)[66]	COx, MAPopt	0.3	34 children, post arrest	MAP below NIRS derived MAPopt is associated with worse outcome.
Lee et al (2014)[65]	HVx	-1/+1	36 children, post arrest	Deviation from ABP with optimal CA may predict poor neurological outcome.
Lovett et al (2018)[68]	TCD Transient hyperaemic response ratio	NS	26 children, post arrest, asphyxia, drowning	Unfavourable outcome had lower cerebral blood flow velocity.<br>Intermittent intact CA was more frequent in those with favourable outcome.
Zipfel et al (2022)[67]	PRx	0.24	19 children, post arrest	Significant doses of impaired CA within 72 h after resuscitation are associated with unfavourable outcome.

PAEDIATRIC STROKE (3)

Authors	***CA Monitoring***	***Cut-off***	***Patient population***	***Results***
Appavu et al (2021)[71]	PRx, wPRx	NA	14 children, AVM	Disturbed CA associated with worse outcome and acquired epilepsy.
Lee et al (2013)[69]	COx<br>HVx	NA<br>-1→+1	6 children, Moyamoya disease	NIRS derived index may identify hemodynamics goals that optimize CA.
Lee et al (2018)[70]	HVx	NA	15 children, Moyamoya disease	Deranged CA during surgery was associated with postoperative transient ischemic attacks in children with bilateral vasculopathy.

OTHER PEDIATRIC CONDITIONS (9)

Authors	*CA Monitoring*	*Cut-off*	*Patient population*	*Results*
Janzarik et al (2021)[72]	Mx; Sx; Dx	NA	12 children brain tumour, 12 healthy controls	Reduced cerebral autoregulation in patients, that did not reach statistical significance.
Jildenstål et al (2021)[74]	Correlation coefficient $CrSO_2$/ABP	NA	15 children under sevoflurane anaesthesia	During sevoflurane anaesthesia CBF seems to be "pressure dependent" indicating a poor efficiency of CA.
Kim et al (2009)[79]	Oxy-Hb /deoxy-Hb	NA	53 healthy children during standing position	Children with abnormal circulatory responses while standing showed a significant reduction of oxy-Hb compared with normal counterparts, suggesting impaired CA in these children.
Ma L et al (2014)[73]	sARI	0.4	32 children DKA<br><br>50 controls	Impaired CA was common early during DKA.
Tontisirin et al (2007)[77]	sARI	0.4	48 children	No gender differences in terms of CA, girls between 4-8 years of age had higher middle cerebral and basilar artery blood flow velocity than age-matched boys.
Vavilala et al (2002)[76]	dARI	NA (0→9)	17 healthy adolescent	Adolescents had significantly lower ARI, higher Vmca than adults.
Vavilala et al (2005)[78]	ARI	0.4	26 children/adolescent	Intact CA but difference in gender between anterior & posterior circulation, girls had higher middle cerebral artery and basilar artery velocity.
Vu et al (2022)[75]	COx	NA	50 children CCH<br><br>100 healthy controls	CA appears to be intact in congenital central hypoventilation patients, however when BP is below the LLA greater hypotension were observed.
Wagner et al (2011)[80]	ΔHb oxygenation /ΔHb volume	NA	24 patients (11 newborns and 13 children)	The correlation between ΔHb oxygenated and ΔCBF after single phenylephrine dose could be a reliable method for CA assessment.

PEDIATRIC AND NEONATAL CARDIAC SURGERY (12)

Authors	*CA Monitoring*	*Cut-off*	*Patient population*	*Results*

Bassan et al. (2005)[90]	MAP-HbD correlation	NA	43 infants undergoing CPB	Found disturbed CA in 51% of subjects on a fluctuating basis, with abnormalities seen during about 15% the studied time.
Brady et al. (2010)[85]	COx	0.4	54 children undergoing CPB	Hypotension was associated with increased COx. The LLA was identified in 77% pt.
Busch et al. (2020)[96]	Microvascular CBF, total Hb concentration	NA	9 children on ECMO	Unable to identify impaired CA.
Cabrera et al. (2018)[88]	HVx	0	16 pts- superior cavopulmonary anastomosis (BCPA)	Elevated ABP post-BCPA is associated with impaired CA, and elevated PAP. Hypotension is not associated with dysregulation.
Easley et al. (2018)[87]	HVx	0.3	57 children CPB	Optimal ABP (48+-11mHg) was identified in all patients and LLA in 82%. There was a significant association between an individual's peak CA and GFAP value.
Joram N et al. (2021a)[93]	COx	0.3	30 children on ECMO V-V, V-A	Relationship between $PaCO_2$ and CA, influenced BP. Low MAP, hypercapnia may disturb CA as it increases LLA.
Joram N. et al. (2021b)[92]	COx	0.3	29 children on ECMO V-V, V-A	Children who developed an acute neurological event had higher COx values and spent more time above the autoregulatory threshold of COx > 0.3.
Ortega et al (2019)[95]	MAP-$CrSO_2$ Wavelet COH	NA	20 children on ECMO<br><br>CA measured in 13 children, 5 controls	They showed a significant relationship between adaptive immune response brain peptides in circulating B and T cells) with acquired brain injury and loss of autoregulation using linear regression.
Smith et al. (2017)[86]	HVx	NA	72 neonates undergoing CPB	Hypothermia & hypotension was associated with a more positive HVx value, but uncertainity if hypotension or hypothermia were the cause for impaired CA.
Tian et al. (2021)[94]	MAP-$CrSO_2$ COH<br><br>And wCOx	NA	148 infants with respiratory insufficiency	The ischemic group showed CA abnormalities associated with low BP and the haemorrhagic group showed abnormalities associated with higher BP. MRI scans were used.
Votava-Smith J. et al. (2017)[89]	MAP-$CrSO_2$ COH	0.5	24 neonates with CHD	Impaired CA in preoperative infants with full-term congenital heart disease.

Zipfel et.al (2022)[91]	COx, HVx,<br><br>ABPopt	COx 0.4<br>HVx 0.3	36 infants after CPB	Optimal MAP identified in 88.9% with the HVx and in 91.7% of children with the COx and LLA could be found in 66.7% and 63.9% of patients, respectively. LLA is higher than the therapeutic goals of patient care reported in the literature.

NEONATAL STUDIES (78)

Authors	***CA Monitoring***	***Cut-off***	***Study population***	***Results***
Alderliesten et al. (2013)[127]	COx	0.5	90 preterm infants (IVH=30, controls=60) aged 0-72h	Altered CA significantly more often before mild/moderate and severe IVH vs. no IVH.
Baerts et al. (2013)[138]	COx	0.5	36 preterm infants (GA 25-29 weeks) aged 0-24h (antenatal tocolytic indomethacin=18, controls=18)	No significant difference in the prevalence of impaired CA.
Beausoleil et al. (2018)[150]	COH and TF gain $CrSO_2$-HR	NA	19 pretem infants <28 weeks GA aged 0-72h (pulmonary haemorrhage /IVH=8; controls=11)	IVH-pulmonary haemorrhage associated with lower cross-correlation, in-phase semblance and TF gain coefficients compared to controls.
Boylan et al. (2000)[158]	MAP-CBFV gradient	NA	25 term & preterm infants (GA 26-42 week)<br><br>(high-risk term infants = 11, high-risk preterm infants =3, term controls =5, preterm controls=7)	Intact CA in term controls without neurologic impairment; altered CA in term and preterm high-risk infants and in preterm controls.

Burton et al (2015)[169]	HVx	ABPopt= lowest HVx	28 term neonates with HIE undergoing TH	ABP<ABPopt duration, deviation and higher ABPopt during rewarming associated with poorer psychomotor outcomes at 2 years.
Caicedo et al (2011a)[107]	MAP-CrSO$_2$, MAP-HbD correlation, COH, partial COH	NA	53 preterm infants (GA <32 weeks)	Positive correlation between higher correlation values and CRIB score. No association with psychomotor outcomes.
Caicedo et al (2011b)[99]	COx<br>MAP-CrSO$_2$ COH	NA	54 preterm infants	No significant differences between CA measured with two different NIRS devices (NIRO300 and INVOS4100).
Caicedo et al. (2012)[102]	COx<br>MAP-CrSO$_2$ COH and TF gain	NA<br>NA	18 preterm infants (mean postmenstrual age 27.9 weeks)	TF gain and COx are most robust methods for CA assessment compared to COH. TF can also assess the causal relationship between MAP and CBF.
Caicedo et al (2013)[139]	COx<br>MAP-CrSO$_2$ COH and TF gain	NA	56 preterm infants (GA 24-31 weeks) aged 0-3 days (labetalol-treated maternal hypertension =21, other treatments =19, controls=16)	Greater CA impairment (higher TF gain) in infants born by labetalol-treated hypertensive mothers.
Caicedo et al. (2014)[131]	Bivariate phase rectified signal averaging (BPRSA) between MAP/HR and CrSO$_2$	NA	9 preterm infants (GA<32 weeks; severe IVH= 5, no IVH=4).	Different BPRSA dynamics between severe IVH infants and controls.
Carrasco et al. (2018)[165]	HVx	ABPopt= lowest HVx	25 term/near-term HIE undergoing TH	ABP<ABPopt duration, deviation and higher ABPopt during rewarming associated with increased brain MRI abnormalities.
Chavez-Valdez et al. (2017)[172]	HVx	ABPopt= lowest HVx	65 term HIE neonates undergoing TH	ABP>ABPopt associated with shorter duration of intubation in boys but longer ventilatory support in girls.<br>ABP<ABPopt related to milrinone administration in girls and longer intensive care stay in boys.

Chen et al. (2022)[163]	MAP-HbD COH<br><br>HVP<br><br>HVx	NA<br><br>NA<br><br>NA	53 term HIE neonates undergoing TH	Altered HVx during TH and MAP-HbD coherence during TH and rewarming associated with brain injury or death.
Chock et al. (2012)[122]	COx	0.5	40 preterm infants <1500 g (PDA ligation=10, indomethacin=12, conservative management = 6, controls=12)	PDA ligation associated with greater CA impairment than conservative/pharmacological management.
Chock et al. (2020)[128]	COx	0.5	103 preterm infants <32 weeks GA and <1250g aged 0-4 days	Impaired CA in 80% of infants with adverse outcome (deceased or developing IVH/periventricular leukomalacia).
Cimatti et al. (2020)[154]	TOHRx	NA	20 preterm infants (IVH=8, controls=12) aged 0-72h	Significantly higher TOHRx before IVH detection compared with controls.
Cohen et al. (2019)[113]	COx	0.5	114 preterm infants (fetal growth restriction=57, matched controls=57) aged 0-72h	Higher COx on day 2 and 3 in fetal growth-restricted neonates vs. controls.
Da Costa et al. (2015)[110]	TOHRx	ABPopt= lowest TOHRx	60 preterm infants <32 weeks GA	TOHRx used to define individual ABPopt.
Da Costa et al (2018)[111]	TOHRx	ABPopt= lowest TOHRx	44 preterm infants <28 weeks GA aged 0-24h[75]	ABP<ABPopt deviations associated with increased mortality and IVH rates.
Da Costa et al. (2019)[132]	COx<br><br>TOHRx	NA<br><br>NA	43 preterm infants (IVH=14, controls=29) aged 0-48h	No difference in mean COx and TOHRx between IVH and no-IVH groups.

Eriksen et al (2015)[103]	COx<br><br>MAP-$CrSO_2$ COH	0.4<br><br>0.5	60 preterm infants <32 weeks GA aged 0-24h	Weak correlation between COx and coherence coefficient.<br><br>COx appeared as a more robust and simpler method to describe CA.
Eriksen et al. (2014)[140]	COx	NA	60 preterm infants <32 weeks GA (13 treated with dopamine) aged 0-24h	Greater CA impairment (higher COx) in association with dopamine treatment.
Fenton et al. (1992)[145]	CO_2-MAP-CBFV changes	NA	94 ventilated preterm infants (≤33 weeks GA)	Altered CA in infants ≤30 weeks GA.
Gilmore et al. (2011)[174]	COx	0.5	23 preterm infants ≤30 weeks GA aged 0-72h	No association between neurologic injury and %time with COx>0.5.
Gilmore et al (2021)[133]	HVx	ABPopt= lowest HVx	63 term HIE neonates undergoing TH	MABP>ABPopt duration on day 3, but not earlier, associated with brain injury.
Govindan et al (2016)[151]	MAP-HbD COH<br><br>HR-HbD COH	0.384	82 term/ preterm infants (GA: 23-41 weeks)-HIE infants=43; CHD=19; preterm undergoing PDA ligation=12; preterm early postnatal period=8.	Poor agreement between MAP-HbD and HR-HbD COH in the left and right hemispheres.<br><br>Baroreflex failure (COH MAP-HR not significant) may affect HR reliability for the detection of altered CA.
Govindan et al (2020)[177]	MAP-HbD COH	NA	68 infants (preterm infants=19; CHD=11; HIE=38).	Ventilator-related CBV fluctuations, sedatives and pCO_2 associated with MAP/HbD; cerebral pressure passivity associated with brain injury development.
Hahn et al (2012)[136]	MAP-$CrSO_2$ COH	0.45 (low frequency)<br><br>0.47 (very low frequency)	60 preterm infants ≤32 weeks' GA aged 0-3 days	Greater CA impairment at decreasing MAP.

Hanigan & Bogner (2010)[176]	MAP-CBF correlation (measured with thermal diffusion)	NA	9 neonates undergone CSF diversion for congenital hydrocephalus	Absence of CA associated with death or severe developmental delay.
Hoffman et al (2019)[126]	COx	0.5	61 preterm infants (GA 23-29 weeks) aged 0-96h	In infants without adverse outcomes, %time with COx>0.5 decreased by postnatal day. Infants with IVH or who died spent more %time with COx>0.5 compared with controls.
Hoffman et al (2021)[118]	COx	NA	61 preterm infants <29 weeks aged 0-96h	Inverse correlation between COx and pCO_2 on day 1.
Howlett et al (2013)[168]	HVx	ABPopt= lowest HVx	24 term HIE neonates undergoing TH	ABP<ABPopt duration during TH and rewarming associated with moderate-severe brain MRI abnormalities.
Huvanandana et al. (2019)[147]	COx<br><br>MAP-$CrSO_2$ COH and cross-correlation	NA	30 preterm infants <34 weeks GA aged 2.6±2.2 days receiving caffeine load	Improved CA (reduced COx and MAP-$CrSO_2$ cross-correlation) following caffeine load administration.
Jayasinghe et al (2003)[119]	Xe-133 CBF-MAP correlation	95% CI >0	16 preterm infants (GA range 24-32 weeks; 5 normotensive, 11 hypotensive) aged 0-96h	Altered CA (MAP-CBF reactivity 1.9% [95% CI 0.8-3.0%)/mmHg) in hypotensive infants.
Kaiser et al (2005)[117]	MAP-CBFV correlation slope	0	43 ventilated preterm infants aged 0-7 days	The autoregulatory slope progressively increases at $PaCO_2 \geq 45$mmHg, suggesting loss of CA.
Kim et al. (2020)[124]	Correlation between systolic, mean, diastolic ABP and CBFV	0	113 preterm infants (GA range 23-29 weeks) aged 0-7 days	Intact CA during systole regardless of PDA size, while during diastole proved better in infants with a large PDA.

Kooi et al. (2020)[123]	COx	NA	27 preterm infants <32 weeks GA undergone PDA ligation	Impaired CA during and up to 12h after surgical PDA ligation.
Kuik et al. (2018)[121]	MAP-cFTOE correlation	-0.3	19 preterm <32 weeks GA undergoing laparotomy due to NEC/spontaneous intestinal perforation	Greater CA impairment during surgery than before/after surgery,<br>Higher $PaCO_2$ associated with a greater risk of CA impairment during surgery.
Lee et al (2017a)[173]	HVx	ABPopt= lowest HVx	75 term HIE neonates undergoing TH	ABP<ABPopt during normothermia associated with liver injury.
Lee et al (2017b)[167]	HVx	ABPopt= lowest HVx	64 HIE neonates undergoing TH	ABP<ABPopt duration and deviation during TH and rewarming associated with increased brain MRI abnormalities.
Lemmers et al (2006)[115]	COx<br>MAP-cFTOE correlation	0.5<br>-0.5	38 preterm infants <32 weeks GA with (n=18) and without respiratory distress (n=20) aged 0-72h	Greater duration of CA impairment in infants with respiratory distress.
Li et al (2016)[148]	COx	NA	44 preterm infants (GA range 27-31) receiving surfactant via less-invasive technique (n=22) or endotracheal tube (n=22)	Impaired CA during and after surfactant administration; shorter effect after less-invasive technique than endotracheal instillation (<5 min vs. 5–10 min).
Li et al. (2023)[161]	HVP	NA	53 HIE neonates undergoing TH	Association between plasma biomarkers of brain injury and dysfunctional CA.
Lightburn et al (2013)[142]	%change of CBFV per MAP	0	15 hypotensive preterm infants <28 weeks GA aged 0-24h.	Pressure-passive CBFV increase as MAP was optimized by dopamine treatment.
Liu et al (2021)[171]	wHVx	ABPopt= lowest wHVx	63 term HIE neonates undergoing TH	ABP>ABPopt with upper MAP=50-60 mmHg during TH may reduce the risk of brain injury.

Martini et al. (2022)[153]	TOHRx	NA	77 preterm infants <32 weeks GA aged 0-72h (IVH=16)	Greater CA impairment associated with haemodynamically significant PDA, ongoing dopamine treatment, increasing mortality scores and high-grade IVH.
Massaro et al (2015)[160]	MAP-HbD COH	NA	36 term or near-term neonates with moderate to severe HIE during TH	Increased time with impaired CA in both cerebral hemispheres associated with higher rates of death or brain injury at MRI.
Massaro et al. (2021)[162]	HVP	0.34	50 term/near-term HIE neonates undergoing TH	HVP >0.34 at 24h of life predicted death or severe brain injury and death or developmental delay.
Menke et al (1993)[97]	MAP-CBFV correlation	NA	16 ventilated preterm infants <33 weeks GA aged 2-8 days	CA capacity of 7.5% (-12.5 to 20.1%) rise in CBFV/1 kPa rise in MABP.
Menke et al (1997)[98]	MAP-CBFV COH	NA	15 ventilated preterm infants (GA range 25-32) aged 1-3 days	Initially reduced CA capacity, with progressive improvement during the monitoring period.
Mitra et al (2014)[152]	TOHRx	NA	31 preterm infants <28 weeks GA aged 0-72h	TOHRx inversely correlated with GA, birth weight and positively correlated with higher CRIB II index.
Munro et al (2004)[109]	MAP-CBF correlation	NA	17 preterm infants <28 weeks GA (12 hypotensive and treated with dopamine) aged 0-2 days	Impaired CA in hypotensive infants before and during dopamine treatment, but not in normotensive infants.<br>A breakpoint at MAP <30 mmHg was identified in untreated infants.
O'Leary et al (2009)[130]	MAP-HbD TF gain	NA	88 preterm 0-5 days<br>(GA range: 23-30 weeks;<br>IVH=37, Early parenchymal echodensities =10, late parenchymal abnormalities=19)	Significant association between altered CA and IVH development.
Pfurtscheller et al. (2022)[116]	COx	NA<br>NA	47 preterm infants (GA ≤36 weeks) with (n=25) and without (n=22)	Significant association between altered CA and need for respiratory support.

	MAP-cFTOE correlation		respiratory support, monitored during the first 15min after birth	
Polavarapu et al (2018)[114]	COx	0.5	46 preterm infants (GA 23-29 weeks) with (=25) and without (=21) abnormal antenatal Doppler aged 0-4 days	Antenatal brain sparing associated with impaired CA at 24- 48h of life and increased risk of severe IVH and/or death.
Pryds et al (1989)[125]	Xe-133 CBF-MAP correlation	95% CI >0	57 preterm infants (GA <35 weeks) aged 0-48h (mild intracranial haemorrhage=9, severe intracranial haemorrhage =10, controls=38)	Significant CA impairment in infants developing severe IVH vs. controls.
Pryds et al (1990)[157]	Xe-133 CBF-MAP correlation	NA	31 term neonates (perinatal asphyxia=19, controls=12) aged 0-24h	Abolished CA and increased CBF in asphyxiated infants who died or developed severe brain damage.
Rhee et al. (2014)[106]	Correlation between systolic, mean, diastolic ABP and CBFV	NA	179 preterm infants (GA 23-33 weeks) aged 0-7 days	Pressure-passive systolic CBFV at lowest GA.<br>Systolic CA capacity improved with increasing GA.<br>Diastolic CA capacity altered in all subjects with minimal GA-related changes.
Richter et al. (2020)[112]	COx	0.5	53 preterm infants <32 weeks GA aged 0-5 days	Greater CA impairment in infants with antenatal brain sparing.
Riera et al. (2014)[100]	Bivariate autoregressive spectral COH (BiAR-COH) MAP-$CrSO_2$	0.57 to detect low superior vena cava flow	54 ventilated preterm infants <31 weeks GA aged 0-4 days	Greater CA impairment predicted severe IVH and mortality.

Riera et al. (2016)[101]	Partial directed COH (PDC) MAP-$CrSO_2$	0.55 to detect low superior vena cava flow	54 preterm infants <30 weeks GA aged 0-2 days	Greater CA impairment predictive of severe IVH and mortality.
Schat et al. (2016)[120]	MAP-cFTOE correlation	NA	28 preterm infants (15 with necrotizing enterocolitis [NEC] and 13 controls)	Impaired CA in 60% of infants with NEC vs. 38% of infants without NEC (not significant).
Solanki & Hoffman (2020)[141]	COx	0.5	61 preterm infants (GA range 24-30 weeks) aged 0-96h	Impaired CA associated with dopamine exposure in a dose-dependent fashion (peak at a concentration of 11–15 µg/kg/min).
Soul et al. (2007)[105]	MAP-HbD COH	0.5	90 preterm infants (GA 23-30 weeks) aged 0-5 days	Impaired CA in 87/90 infants and associated with low GA and BW, systemic hypotension, and maternal hemodynamic factors.
Sunwoo et al. (2022)[149]	RiSC (average sliding correlation index between scalp blood flow and CBF, using DCS)	NA	19 preterm infants (GA <28 weeks, IVH=9) aged 2-5 days	Higher RiSC in severe IVH occurring within 72 h of life.
Tekes et al (2015)[166]	HVx	ABPopt= lowest HVx	27 HIE neonates undergoing TH	ABP<ABPopt deviation during TH and rewarming associated with increased brain MRI abnormalities.
Thewissen et al (2018)[144]	MAP-$CrSO_2$ COH	0.3	22 preterm infants <37 weeks receiving propofol for intubation	Impaired CA in one patient during propofol-related hypotension and in 5 patients with normal-to-raised MABP.
Thewissen et al (2021)[143]	MAP-$CrSO_2$ COH	NA	89 preterm infants <28 weeks GA (hypotensive = 36, 13 treated with dopamine, 16 with placebo) aged 0-72h	Impaired CA in hypotensive vs. normotensive infants.<br>No CA difference between dopamine and placebo.

Tian et al. (2016)[159]	MAP-CrSO$_2$ COH	NA	9 term neonates with HIE during TH	Increased in-phase and anti-phase coherence associated with worse outcomes (death, brain MRI injury, abnormal neurodevelopment).
Tian et al. (2021)[175]	MAP-HbD COH	NA	14 term and near-term neonates with mild HIE	No CA difference between brain areas with and without MRI lesions.
Tsuji et al (2000)[129]	MAP-HbD COH	0.5	32 preterm infants (GA 23-31 weeks) aged 0-3 days	Impaired CA in 53% of the study population, of which 47% developed severe brain lesions. Among infants with intact CA, only 13% developed severe brain lesions.
Van de Bor et al (1991)[108]	MAP-CBFV correlation	NA	48 preterm infants ≤32 weeks GA aged 12-72h	Preserved CA for MAP ranging from 31 to 40 mmHg.
Vedrenne-Cloquet et al (2019)[146]	MAP-cFTOE correlation	NA	22 preterm and term infants receiving atropine-propofol (n=11) vs. atropine-atracurium-sufentanil (n=13) for intubation.	No correlation between MAP and cFTOE in both pharmacological groups.
Verma et al (2000)[137]	MAP-CBFV correlation slope	NA	62 term and preterm neonates aged 2-72h	MAP-CBFV slope decreased at increasing GA.<br>No significant correlations with PaCO$_2$ and postnatal age.
Vesoulis et al. (2016)[104]	MAP-CrSO$_2$ TF gain	NA	62 infants <28 weeks GA aged 0-72h	Increasing CA capacity for increasing GA and birth weight z score; weaker CA capacity in African-American infants and in association with IVH.
Vesoulis et al (2018)[170]	COx	ABPopt= positive to negative COx shift	16 term/near term HIE neonates undergoing TH	ABP>ABPopt duration on day 3, but not earlier, associated with brain injury.
Vesoulis et al. (2019)[134]	MAP-CrSO$_2$ TF gain	NA	45 preterm infants <28 weeks GA (delayed cord clamping, n=15; controls, n=30) aged 0-24h	Delayed cord clamping associated with improved CA capacity; improved CA capacity associated with decreased IVH risk.
Wong et al. (2008)[135]	MAP-CrSO$_2$ COH	0.5	24 preterm infants <32 weeks' GA aged 0-2 days	Impaired CA associated with lower GA, BW, MAP, and higher mortality.

Abbreviations: *ABP arterial blood pressure; ABPopt ABP optimum; BPCA bidirectional cavopulmonary anastomosis; BW birth weight; CA cerebral autoregulation; CBF cerebral blood flow; CBFV cerebral blood flow velocity; cFTOE, cerebral fraction of tissue oxygen extraction; CHD congenital heart disease; COH coherence; COx cerebral oxygenation index;* CO_2 *carbon dioxide; CPB cardiopulmonary bypass;* $CrSO_2$ *cerebral regional oxygen saturation; dARI dynamic autoregulatory index; DATACAR dynamic adaptive target of active cerebral autoregulation; DCS diffuse correlation spectroscopy; DKA diabetes ketoacidosis; ECMO V-A Veno-arterial extracorporeal membrane oxygenation; ECMO V-V Veno-venous extracorporeal membrane oxygenation, GA gestational age; GFAP glial fibrillary acidic protein; HbD oxygenated haemoglobin; HIE hypoxic-ischemic encephalopathy; HR heart rate; HVx haemoglobin volume reactivity index; HVP haemoglobin volume phase index; IVH interventricular haemorrhage; LAx low frequency autoregulation index; LLA lower limit of autoregulation; Mx mean flow index; MAP mean arterial pressure; MAPopt MAP optimum; MRI magnetic resonance imaging; mTBI mild TBI, moTBI moderate TBI; NA not available; NIRS near infrared spectroscopy; PAP pulmonary artery pressure; PAx pulse amplitude index;* $PaCO_2$ *partial pressure carbon dioxide; PDA patent ductus arteriosus; PI Pulsatility index; PRx pressure reactivity index; pt patient; RoR rate of autoregulation; sTBI severe TBI; TBI traumatic brain injury; TCD transcranial Doppler sonography; TF transfer function; TH therapeutic hypothermia; TOHRx tissue oxygenation heart rate index; wCOx wavelet COx; wPRx wavelet PRx; Xe xenon.*
